# *Escherichia coli* spheroplasts in a Croatian patient misclassified by two urine sediment analysers as erythrocytes: case report

**DOI:** 10.11613/BM.2019.030801

**Published:** 2019-10-15

**Authors:** Ana Nikler, Vanja Radišić Biljak, Helena Čičak, Nikolina Marić, Danijela Bejuk, Jose Antonio Tesser Poloni, Ana-Maria Simundic

**Affiliations:** 1 Department of Medical Laboratory Diagnostics, University Hospital “Sveti Duh”, Zagreb, Croatia; 2 Department of Emergency and Intensive Medicine, University Hospital “Sveti Duh”, Zagreb, Croatia; 3 Department of Clinical Microbiology and Hospital Infections, University Hospital “Sveti Duh”, Zagreb, Croatia; 4 Carlos Franco Voegeli Clinical Analysis Laboratory, Santa Casa de Misericórdia de Porto Alegre, Porto Alegre, Brazil; 5 Universidade do Vale do Rio dos Sinos, São Leopoldo, Brazil; 6 Controllab, Rio de Janeiro, Brazil; 7 Faculty of Pharmacy and Biochemistry, University of Zagreb, Zagreb, Croatia

**Keywords:** spheroplasts, bacterial forms, urinalysis, misclassification

## Abstract

**Introduction:**

It has already been reported that subinhibitory concentrations of β-lactam antibiotics can cause abnormal changes of bacterial forms, such as spheroplasts. Herein we report a case of Croatian male patient with *Escherichia coli* spheroplasts present in urine after treatment with tazobactam, on the tenth day of hospitalization. The aim of this report is to emphasize the inability of imaging based automated urine analysers to recognize some relatively uncommon forms of bacterial presentation in urine sediment.

**Materials and methods:**

During routine urine analysis, unusual particles were observed in patient urine. Urine sediment was examined by two urine analysers: Atellica 1500 (Siemens, Germany) and Iris iQ200 (Beckman Coulter, USA). Additionally, urine was sent for culture testing to Microbiology department.

**Results:**

Both urine analysers didn’t indicate presence of bacteria in urine sediment. Unusual particles observed on the tenth day were classified as erythrocytes by both instruments. Dipstick test showed blood trace and microscopic analysis revealed bacteria in urine. Urine culture was positive for *Escherichia coli.* Careful examination of urine sediment has confirmed that shapes present in urine were abnormal bacterial forms called spheroplasts.

**Conclusions:**

Imaging based automated urine analysers are not able to recognize bacterial spheroplasts in urine sediment misclassifying it as erythrocytes. Microscopic examination remains the gold standard for urines with blood trace or negative blood, in which erythrocytes are reported by urine analyser in urine sediment. Failure to identify and follow up such cases may lead to inaccurate treatment decisions and puts patient safety at risk.

## Introduction

The bacterial shape is determined by the structure of its cell wall, which can be easily modified to survive environment conditions (*1*). The peptidoglycan and various cytoskeletal proteins located in the bacterial cell wall (*e.g.* MreB, crescentin, FtsZ), play a major role in shaping the bacterial cells and changing its form (*2*-*5*). These abnormal bacterial forms are called cell wall deficient (CWD)-forms which have been classified as protoplasts and spheroplasts, depending on the degree of the loss of cell wall components (*2*). Protoplasts have completely lost the cell wall while spheroplasts usually have partly maintained cell wall. Therefore, spheroplasts are more likely to survive untoward conditions. It has been observed that low concentrations of antibiotics can induce the change of bacterial form. For example, β-lactam antibiotics produce shape changes at concentrations significantly below those necessary to stop bacterial multiplication (*6*-*8*). The β-lactams act by binding to penicillin-binding proteins (PBPs) which induce the peptidoglycan synthesis. Three mechanisms through which β-lactams act on bacterial growth are rapid lysis, production of spherical cells or filamentation (*9*). Subinhibitory concentrations of these antibiotics result in formation of abnormal, spherical forms that can be observed in the urine of treated patient (*10*).

Here we report a case of *Escherichia coli* spheroplasts present in the urine of a patient treated with tazobactam. The aim of this report is to emphasize the inability of imaging based automated urine analysers to recognize bacterial spheroplasts.

## Materials and methods

### Case history

A 48-year-old male patient in vegetative state, cachectic and with tracheostoma was admitted to the Emergency Department of University Hospital “Sveti Duh” (Zagreb, Croatia) because of the deterioration of his health status, decubitus ulcers and severe hyponatremia. Patient basal condition (*i.e.* vegetative coma) was the consequence of drug intoxication (promazine, citalopram, ibuprofen) that occurred two years ago. Additionally, his past medical history included sepsis, cardiorespiratory arrest, kidney failure, bronchopneumonia, left-sided pleural effusion, urosepsis and brachycardia. Previous therapy included levothyroxine, pantoprazole, bisoprolol, sodium valproate and clonazepam. Patient was hospitalized and his decubitus ulcers were treated intravenously with fluoroquinolone antibiotic ciprofloxacin. On the sixth day, the abundant secretion from tracheostoma was noticed and therefore the microbiological analysis of tracheostomic sample was made, which resulted in isolation of *Proteus mirabilis* and *Klebsiella aerogenes*. Because of that bacterial infection, the eight-day treatment with ciprofloxacin was stopped and the patient was treated with tazobactam, β-lactam antibiotic.

During the first 10 days of hospitalization, three urine samples of this patient were sent to our laboratory for qualitative urine dipstick and sediment analysis. Urine samples were collected by catheter. Urine was each time analysed by two imaging based automated urine analysers: Atellica 1500 (Siemens, Germany) and Iris iQ200 (Beckman Coulter, USA). These two automated urine analysers differ respective to their method of the analysis of urine sediment: Iris iQ200 is an image-based analysis system, which creates images of the laminar urine flow with a digital camera coupled with a microscope, whereas Atellica 1500 is an automated digital microscopy urine sediment analyser. Original urine test strips were used in urinalysis (*e.g.* they were from the same manufacturers as the analysers). These two analysers have different reporting categories for urine sediment results. Results for particle analysis in urine were provided in absolute numbers and in categories declared by both manufacturers.

First two urine samples were analysed on the third and sixth day of the hospitalization. During that time the patient was treated with ciprofloxacin. The third urine sample arrived on the tenth day of the hospitalization when the patient was treated with tazobactam instead of ciprofloxacin. During the routine laboratory urine analysis, we found some unusual elements in urine sediment that intrigued us to investigate this further. Additional investigation included urine culture testing in the Microbiology department.

Publication of this case report was approved by the Ethics committee of University hospital “Sveti Duh” (Zagreb). Since the patient was unconscious, informed consent has been obtained from a family member. “

## Results

**Figure 1:**
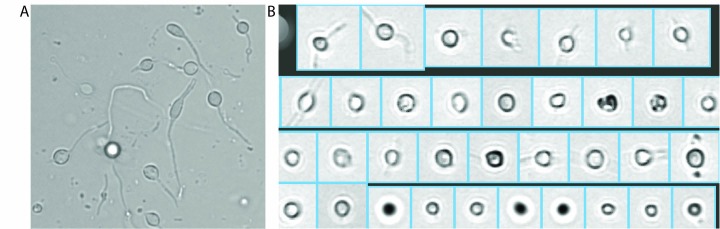
*Escherichia coli* spheroplasts found in native urine sample analysed on Atellica 1500 (A) (400x magnification) and Iris iQ200 (B) urine analyser.

Results of qualitative urinalysis (urine dipstick and urine sediment), as well as urine culture results are showed in Table 1. On the third and the sixth day, urinary sediment examination did not reveal uncommon particles. However, in the third urine sample received on the tenth day, unusual shapes were observed. Both analysers classified the unusual shapes as erythrocytes. Urine culture on the sixth and tenth day was positive pointing to the obviously inefficient antibiotic treatment in this patient. Careful examination of microscopic images captured by both urine analysers on the tenth day, has confirmed the presence of bacterial spheroplasts in patient urine (Figure 1).

**Table 1:** Results of qualitative urinalysis and urine culture of a patient with spheroplasts

**Days**	**DAY 3**			**DAY 6**			**DAY 10**		
**Therapy**	ciprofloxacin			ciprofloxacin			tazobactam		
**Analysis**	**Iris**	**Atellica**	**Urine culture**	**Iris**	**Atellica**	**Urine culture**	**Iris**	**Atellica**	**Urine culture**
**Blood** **(test strips)**	trace	negative	N/A	trace	trace-lysed	N/A	trace	trace-lysed	N/A
**Nitrites** **(test strips)**	+	positive	N/A	negative	negative	*Klebsiella pneumoniae,* *Pseudomonas aeruginosa,* *Acinetobacter baumannii* (> 10^5^)	negative	negative	*Escherichia coli* (> 10^4^)
**Bacteria** **(/µL)**	135	1744		24	247		26	995	
**Bacteria** **rods (/µL)**	N/A	236		N/A	3.52		N/A	179	
**Bacteria cocci (/µL)**	N/A	1508		N/A	244		N/A	816	
	RBC 2 (neg.)	RBC < 4 (neg.)		RBC 3 (neg.)	RBC 58 (1+)		RBC 48 (1+)	RBC 77 (2+)	
	WBC 7 (neg.)	WBC 8 (neg.)		WBC 1 (neg.)	WBC 56 (1+)		WBC 11 (neg.)	WBC 34 (1+)	
	EPI 1 (neg.)	HYA 0.44 (neg.)	N/A	EPI 2 (neg.)	HYA 3 (1+)	N/A	YEA 3 (neg.)	WBCc 1 (neg.)	N/A
**Urine sediment**	MUC 2 (neg.)	MUC 25 (neg.)		MUC 5 (neg.)	PAT 1 (neg.)		EPI 2 (neg.)	HYA 0.4 (neg.)	
					NEC 1.8 (neg.)		MUC 25 (neg.)	YEA 106 (1+)	
					EPI 2.6 (neg.)			MUC 56 (neg.)	
					MUC 90 (neg.)				
RBC - erythrocytes. WBC - leukocytes. WBCc - clump of leukocytes. HYA - hyaline cast. PAT - pathological cast. NEC - nonsquamous epithelial cell. EPI- squamous epithelial cell. MUC- mucus. Reference values for nitrites and blood are negative. N/A - not applicable. neg-negative.									

## Discussion

With this case we demonstrate the inability of imaging based automated urine analysers to recognize the unusual bacterial forms (spheroplasts) present in the urine sample of a hospitalized patient treated with subinhibitory concentrations of tazobactam, β-lactam antibiotic.

Changes in the structure of bacterial cell wall present a surviving mechanism which helps bacteria to survive in the presence of antibiotics (*2*, *6*, *10*, *11*). Most of the studies which have reported such changes in the structure of the bacterial wall were based on Gram stained urine and phase-contrast and electron microscopy images. However, nowadays most routine laboratories use optical microscopy of unstained urine for urine sediment analysis. If laboratory professionals involved in the urine sediment examination are not able to recognize these unusual bacterial forms, they might be misinterpreted as erythrocytes or fungi. Poloni *et al.* have published a case report of spheroplasts present in unstained urine sample of a patient that had recurrent episodes of urinary tract infection (*12*). In the urine culture of that patient, *Klebsiella pneumoniae* ESBL was identified. The authors have concluded that spheroplasts were most probably associated with subinhibitory concentrations of β-lactam antibiotic with which this patient was treated with.

Spheroplasts observed in our patient case ranged different in size, including shapes similar to small bacteria to shapes as large as erythrocytes. Although spheroplasts may appear to look like erythrocytes at the first sight, careful examination can easily reveal their real origin. The main difference between erythrocytes and spheroplasts are filaments of varying lengths present in spheroplasts. Spheroplasts do not have biconcave shape characteristic for erythrocytes and they can be present as round but also as elongated form. Small round spheroplasts can also be misclassified as fungi but long spheroplast filaments differ from hyphae. Hyphae have septate branching structure while spheroplasts consist of only one or two filaments that extend from one bacterial cell (*13*). Both urine analysers that we used in our laboratory to examine this urine sample were not able to recognize spheroplasts and have mistakenly classified them as erythrocytes. This false positive blood result of urine analysis and non-recognition of bacterial spheroplasts may lead to wrong medical diagnosis and treatment decisions that affect patient safety and outcome.

For a definitive confirmation of our findings, some kind of a confirmatory method (*e.g.* molecular analysis) is preferred. Unfortunately, we were not able to run any of the confirmatory methods and we recognize this as a possible limitation of this study.

A take-away lessons learned from this case for us was that subinhibitory concentrations of β-lactam antibiotics may lead to the formation of abnormal, spherical bacterial forms in urine (spheroplasts) which may not be easily detectable and recognized by imaging based automated urine analysers. Bacterial spheroplasts could be misclassified by urine analysers as erythrocytes, while laboratory staff could mistakenly classify them as fungi during manual sediment examination.

To overcome this problem, laboratory staff should pay extra attention when examining urines with blood trace, in which bacteria and erythrocytes are reported by urine analyser in urine sediment. Furthermore, finding of highly positive erythrocytes in urine sediment in the absence of positive dipstick for blood is unusual and should always be further investigated. Obviously, careful examination and communication with clinicians and clinical microbiologists is a key for proper management of such cases.
